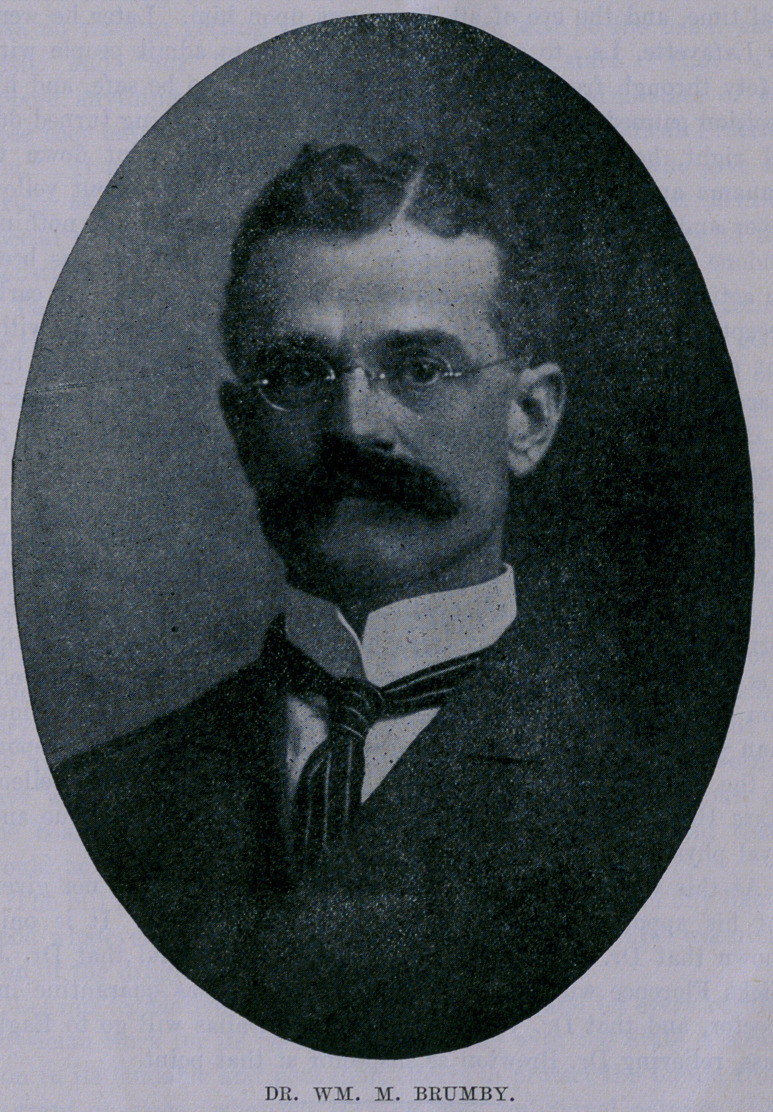# Texas’ New Health Officer and Surgeon General

**Published:** 1907-01

**Authors:** 


### TEXAS' NEW HEALTH OFFICER AND SURGEON GENERAL.

---

We present herewith the picture of Dr. Wm. M. Brumby of Houston, who has been appointed by Governor Campbell to succeed Dr. Geo. R. Tabor as State Health Officer and ex-officio Surgeon General of Texas; and append a brief biographical sketch.

Dr. Brumby was born in Delhi, La., in 1866. At an early age he was sent to the public schools, and continued to the completion of the course, and finished his education at the University of Alabama at Tuscaloosa. Later he went to the medical department of Tulane, in 1889. From there he returned to Delhi, his home, and took

DR. WM. M. BRUMBY.

up the practice of medicine. Thence he went to Houston, and in 1898 he was chosen a member of the Houston Board of Health. About that time he, at the request of Dr. Blunt, State Health Officer, went to New Orleans to investigate the feasibility of bringing certain freights through the quarantine under proper safeguard. He decided that it could be done, and his action in the matter was indorsed and business went forward. He was Assistant Health Officer under Dr. Massie in 1899, in which position he was very active. In 1900 he was elected City Health Officer, and met the responsibilities with much credit to himself. That was a critical time, and the eye of all Texas was upon him. Later he went to Lafayette, La., to see if it were possible to admit people with safety through from Louisiana. He decided it to be safe, and his decision panned out good. These several matters having turned out all right, he, for the better posting of himself, went down to Panama and Central America to find out still more about yellow fever and its treatment under all conditions, and he left nothing undone to accomplish his purpose. His professional life has been an active one, especially in connection with yellow fever. He early accepted the mosquito theory and went after the stegomyia without gloves. His study of yellow fever and its environments has placed him among the first authorities of the land on that subject.

He settled in Houston eleven years ago. He was married at Cameron in 1891, and has a wife and two children.

A few years ago Houston was considered one of the most unhealthy cities in all Texas. Today the mortuary reports compare favorably with any city in the State, and Houston has more miles of sanitary sewers. Much credit is due Dr. Brumby for these reforms and all matters pertaining to public health and sanitation. His work in securing food inspection and measures for the prevention of tuberculosis and other matters of more concern at all times than yellow fever, is set forth and is evidenced by his annual report to the city council in 1902. Dr. Brumby will make an excellent State Health Officer, and should have the support of all true and loyal physicians.

At this writing (January 18) the Governor-elect has not given out his appointments to the several medical offices. It is only known that Dr. Brumby will succeed Dr. Tabor, and that Dr. J. Hicks Florence will be retained at Brownsville as quarantine inspector, and that Dr. V. P. Armstrong of Dallas will go to Eagle Pass, relieving Dr. Brewton as inspector at that point.

## Figures and Tables

**Figure f1:**